# The Use of a Solid Bismuth Microelectrode for Vanadium Quantification by Adsorptive Stripping Voltammetry in Environmental Water Samples

**DOI:** 10.3390/molecules27072168

**Published:** 2022-03-27

**Authors:** Malgorzata Grabarczyk, Marzena Adamczyk, Edyta Wlazlowska

**Affiliations:** Department of Analytical Chemistry, Institute of Chemical Sciences, Faculty of Chemistry, Maria Curie-Sklodowska University, 20-031 Lublin, Poland; malgorzata.grabarczyk@mail.umcs.pl (M.G.); edyta.wlazlowska@onet.pl (E.W.)

**Keywords:** miniaturized sensors, solid bismuth microelectrode, vanadium, voltammetry analysis, cupferron

## Abstract

This paper presents for the first time the use of an environmentally friendly solid bismuth microelectrode for the voltammetric quantification of V(V) in natural water samples. These studies were designed to replace the film bismuth electrode that had been introduced to eliminate the conventional sensors based on highly toxic mercury. In the proposed procedure, V(V) is preconcentrated at the solid bismuth microelectrode surface via the formation of electroactive complexes with cupferron from a solution of 0.1-mol L^−1^ acetate buffer, pH = 4.6 at a potential of −0.4 V. The linearity of the calibration graph is in the V(V) concentration range from 8 × 10^−10^ to 1 × 10^−7^ mol L^−1^ with a preconcentration time of 1 min. The limit of detection (calculated as 3 σ) is 2.5 × 10^−10^ mol L^−1^ for a preconcentration time of 1 min. It was also demonstrated that significant improvement in analytical parameters was achieved as a result of the activation of the solid electrode surface at a potential of −2.5 V for 2 s. The developed procedure is highly selective for the presence of foreign ions and organic compounds in tested samples. The accuracy of the recommended procedure was checked using SPS-WW1 waste water-certified reference materials of a complex composition, in which the concentration of V(V) determined by the proposed method was 95.1 ± 1.6 ng mL^−1^. Moreover, in keeping with the outlined procedure, river, tap and rain water samples were analyzed without any pretreatment, and recovery values from 96% to 106% were obtained.

## 1. Introduction

A solid bismuth microelectrode is a miniaturized sensor that was introduced to the stripping analysis of both metal ions and organic compounds by Geca et al. in 2020 [1,2]. In this novel electrode, melted metallic bismuth is placed within a glass capillary whose thickness and diameter are 5 mm and 25 µm, respectively. Everything is enclosed in a miniaturized casing made of PEEK. Due to this design of the electrode, it is not necessary to add toxic bismuth ions to the supporting electrolyte during the voltammetric measurements. The presence of toxic bismuth ions in a voltammetric cell is indispensable if the working electrode used is a bismuth film electrode generated in situ. Thus, the use of the solid bismuth microelectrode simplifies the procedure and, at the same time, makes it more friendly to the laboratory environment. This is a tremendous advance in the field of electrochemical methods. Moreover, thanks to the use of this microsensor, it is possible to determine the analyte at the nanomolar level from diluted solutions, as well as nonaqueous samples. It should also be emphasized that the use of a microelectrode as a working electrode gives the possibility of miniaturization of the measuring system, as well as making it possible to carry out the analysis at the location of the sampling. Besides, since the capacitive current in the microelectrodes is limited to a minimum, the use of miniaturized sensors allows obtaining a more favorable signal-to-noise ratio compared to the use of classic macroelectrodes [1,2,3].

So far, the most frequently used working electrodes in stripping voltammetry were electrodes modified with films of various metals that displaced mercury electrodes. The first mercury-free electrode of this type was the bismuth film electrode (BiFE), introduced to laboratory work in 2000 by a group of scientists led by Prof. J. Wang [4]. Another lead-modified electrode (PbFE) was developed in 2005 by the research team of Prof. Mieczysław Korolczuk at the helm [5]. The collaboration of scientists from the Czech Republic and Slovenia resulted in the development of another eco-friendly antimony film electrode (SbFE) in 2007 [6]. The preparation of the above-mentioned film electrodes consists in covering the solid electrode, which is the electrode substrate, with a thin metal film obtained electrochemically from the salt of a given element. Although these electrodes are less toxic than mercury electrodes, it is necessary to use heavy metal salts that are also not completely safe for the environment. However, for the production of novel solid bismuth microelectrodes, very small amounts of the electrode material in the form of molten bismuth are needed, and they are additionally closed in a tight miniaturized housing.

Stripping voltammetry is one of the most sensitive and, at the same time, cheapest methods used in trace analysis. The high sensitivity of striping voltammetry results from the two-stage course of the analytical measurement. In the first stage, called preconcentration, accumulation of the substance on the surface of the working electrode takes place at a constant electrode potential. Thanks to this step, the concentration of the analyte accumulated on the electrode with a relatively small surface area becomes much higher than in the solution. The preconcentration process may take place as a result of electrolysis (anodic striping voltammetry—ASV), adsorption (adsorptive striping voltammetry—AdSV) or an electrode reaction leading to the formation of a sparingly soluble compound on the electrode surface (cathode striping voltammetry—CSV). The subsequent stripping step is carried out by applying a varying potential to the electrode. This is the recorded stage, during which an analytical signal in the form of a voltammetric peak is obtained. The stripping stage occurs most often through oxidation, reduction or, less frequently, desorption of the accumulated substance. Out of the voltammetric techniques mentioned above, the AdSV method gives the possibility of obtaining the lowest detection limits due to the use of a complexing agent forming an electroactive metal-complexing agent capable of accumulating on the electrode surface. To date, many adsorptive stripping voltammetric procedures have been developed for the trace quantification of V(V) [7,8,9,10,11,12,13,14,15,16,17,18,19]. The analytical parameters of all those procedures are compared in Table 1. These are all vanadium determination procedures by the AdSV method that, to our knowledge, have been published in the literature. They were used for the determination of vanadium(V) mainly in real water samples [7,9,10,11,12,13,14,15,16,17,18,19] and in food samples [7,12]. All of them are based on the adsorptive accumulation of the V(V) complex exploiting different complexing agent such as cupferron [7,8,14,19], chloranilic acid [9,16,18], catechol [10], dihydroxynaphthalene [11], chromoxane cyanine R [12], dihydroxybenzaldehyde [13], pyrogallol [15] and alizarin violet [17]. Unfortunately, the majority of those procedures are based on the use of mercury-containing electrodes, such as hanging mercury drop electrodes (HMDEs) [7,8,9,10,11,12,13], mercury film electrodes (MFEs) [14,15] and renewable mercury film silver-based electrodes (Hg(Ag)FEs) [16]. The use of the HMDE electrode and chloranilic acid as a complexing agent of V(V) in the procedure [9] guarantees the lowest detection limit of 9 × 10^−12^ mol L^−1^. On the other hand, the use of the same electrode and the chromoxane cyanine R is associated with obtaining a much higher limit of detection equal to 1 × 10^−7^ mol L^−1^ [12]. The widest ranges of linearity from 2 × 10^−9^ to 2 × 10^−6^ mol L^−1^ and from 2.5 × 10^−10^ to 1 × 10^−7^ mol L^−1^ were obtained in the procedures from References [7,16], respectively. The other ones involve the use of an acetylene black paste electrode (ABPE) [17], as well as metal film electrodes such as a bismuth film electrode (BiFE) [18] and a lead film electrode (PbFE) [19]. The detection limits for these procedures range from 3.2 × 10^−10^ [19] to 3.9 × 10^−9^ mol L^−1^ [18]. These procedures are suitable for the determination of vanadium concentrations in environmental water samples ranging from 8 × 10^−10^ [17] to 5 × 10^−7^ mol L^−1^ [18]. As is known, the preparation of the above electrodes involves the introduction of toxic heavy metal salts into the supporting electrolyte. Therefore, in this paper, we first discuss the usage of solid bismuth microelectrodes for the voltammetric determination of trace vanadium. As mentioned earlier, the use of this electrode is currently the friendliest toward a laboratory environment.

Why is it so important to determine vanadium at a trace concentration in environmental water samples? As a matter of fact, every day, large amounts of vanadium compounds are released into the environment in the form of deadly PM2.5 and PM10 dust. It is a result of the combustion of coal, wood and oil, as well as a consequence of the generation of car exhaust fumes. These dusts are aerosols suspended in the air, especially negatively affecting the respiratory system. They cause breathlessness and even contribute to the development of lung cancer. Moreover, they increase the risk of heart attack and stroke. Vanadium dust can also damage the kidneys, skeletal structures, and gastrointestinal mucosa [20,21]. Harmful dust is introduced into water in the environment, along with rain or other atmospheric precipitation and, hence, the need to develop sensitive yet simple and cheap procedures for the determination of trace amounts of vanadium in environmental water samples.

## 2. Materials and Methods

### 2.1. Apparatus

A µAutolab analyzer (Eco Chemie, Utrecht, The Netherlands) was applied for all voltammetric experiments. The conventional three-electrode quartz cell was utilized, which was composed of a solid bismuth microelectrode as a working electrode, an Ag/AgCl/saturated NaCl as a reference electrode and platinum wire as an auxiliary electrode. Details of the design of the solid bismuth microelectrode are given elsewhere [1]. Before each series of measurements, the working electrode was carefully polished in a figure eight motion on 2500 silicon carbide paper and sonicated for 30 s in an ultrasonic bath (Sonic-3, Polsonic, Poland) to remove any polishing residue. Furthermore, the working microelectrode was electrochemically activated before each measurement at a potential of −2.5 V for 2 s.

### 2.2. Reagents

A 1-mol L^−1^ acetate buffer (prepared from Suprapur CH_3_COOH and NaOH obtained from Merck) was applied as a supporting electrolyte. Cupferron (Merck) was used to prepare a 1 × 10^−2^-mol L^−1^ solution acting as a complexing agent for V(V). Working solutions of V(V) were prepared every day by diluting a vanadium standard solution (Merck, 1 g L^−1^). The certified reference material SPS-WW1 waste water (batch 111) (concentration of V(V) equal to 100.0 ± 0.5 ng mL^−1^) was obtained from Spectrapur Standards AS (Oslo, Norway) and used to check the practical suitability of the proposed procedure. To check the selectivity of this procedure, working solutions of a number of foreign ions were used; they were prepared by diluting their standard solutions (1 g L^−1^) to a concentration of 1 × 10^−4^ mol L^−1^ with 1 × 10^−2^ mol L^−1^ of HNO_3_. Additionally, the procedure was tested for sensitivity to Triton X-100 (a representative of surfactants obtained from Fluka) and sodium humic acid (HA) (a representative of humic substances obtained from Aldrich).

### 2.3. Standard Procedure of Measurements

The quantitation of vanadium in environmental water samples was carried out using differential pulse adsorptive stripping voltammetry (DP AdSV) in the manner described below. An appropriate volume of the standard V(V) solution (in the case of optimization studies) or the analyzed sample solution was inserted into the voltammetric cell, and then, 1 mL of 1-mol L^−^^1^ acetate buffer (pH 4.6) and 1 mL of 1 × 10^−2^-mol L^−1^ cupferron were added. After that, the prepared solution was made up of a volume of 10 mL with triply distilled water. The experiments were performed for non-deaerated solutions. The DP AdSV procedure was carried in an unbroken sequence, with the following subsequent stages: (1) brief electrochemical activation (bismuth oxides that may form on the electrode surface are reduced to a metallic form), applying an activation potential of −2.5 V for 2 s; (2) adsorptive preconcentration of the V(V)–cupferron complex onto the microelectrode surface at an accumulation potential of −0.4 V for 60 s with stirring and (3) after 5 s of the rest time, the DP voltammogram was registered by changing the potential values from −0.45 to −0.80 V with a step potential of 3 mV, a pulse amplitude of 30 mV and a pulse period time of 20 ms.

## 3. Results and Discussion

### 3.1. Optimization of Supporting Electrolyte Composition

**Figure 1:**
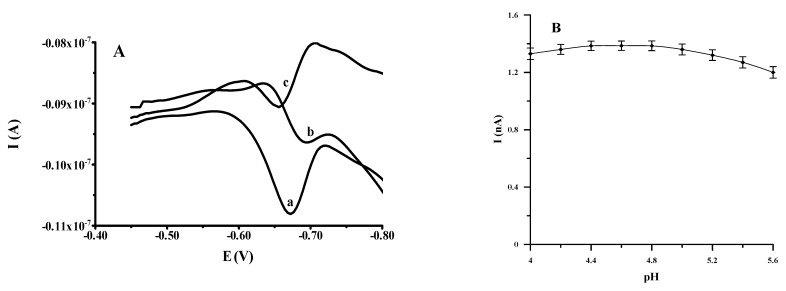
The AdSV voltammograms of 1 × 10^−8^ mol L^−1^ V(V) obtained in the solution containing: 0.1 mol L^−1^ acetate buffer (a), 0.1 mol L^−1^ Britton-Robinson buffer (b) and 0.1 mol L^−1^ phosphate buffer (c) (**A**). The effect of pH of 0.1 mol L^−1^ acetate buffer on the AdSV voltammetric response of 1 × 10^−8^ mol L^−1^ V(V) (**B**). The accumulation potential and time were −0.4 V and 60 s, respectively.

In all previously published procedures for the determination of vanadium in the form of complexes with cupferron, an acidic medium was utilized, which was provided by an appropriate buffer solution. For this purpose, the authors used a phosphate buffer pH = 4.8 [7] and **a** Britton–Robinson buffer pH = 4.0 [8], as well as an acetate buffer pH = 4.6 [14] and pH = 5.6 [19]. Accordingly, our research was devoted to examining the effects of the supporting electrolytes, such as acetate, Britton–Robinson and phosphate buffer, for the voltammetric response of vanadium(V). Both the height of the peak and its shape were taken into consideration during choosing the supporting electrolyte. Out of these buffers, the acetate buffer provided the most promising signal (Figure 1A). Thus, the impact of the acetate buffer pH on the differential pulse signal of V(V) in a solution comprising 1 × 10^−8^-mol L^−1^ V(V) and 1 × 10^−3^-mol L^−1^ cupferron was investigated in a pH range between 4.0 and 5.6 (Figure 1B). It turned out that, in the pH range from 4.4 and 4.8, the peak current of vanadium was at the maximum value. Given the obtained results and good buffering capacitance, a pH value of 4.6 was chosen for further research. 

Afterwards, different concentrations of acetate buffer pH = 4.6 in a range of 0.05–0.20 mol L^−1^ were tested at fixed vanadium and complexing agent concentrations. However, it was found that the signal of V(V)–cupferron reduction was similar over the whole examined concentration range, thus indicating that the studied supporting electrolyte concentrations did not impact the V(V)–cupferron complex formation, and as presumed, the pH of the supporting electrolyte is a strategically valid factor affecting complex formation. To gain better stabilization of the pH of the analyzed solution, a concentration of 0.1 mol L^−1^ was adopted for the whole study.

### 3.2. Optimization of Cupferron Concentration

**Figure 2:**
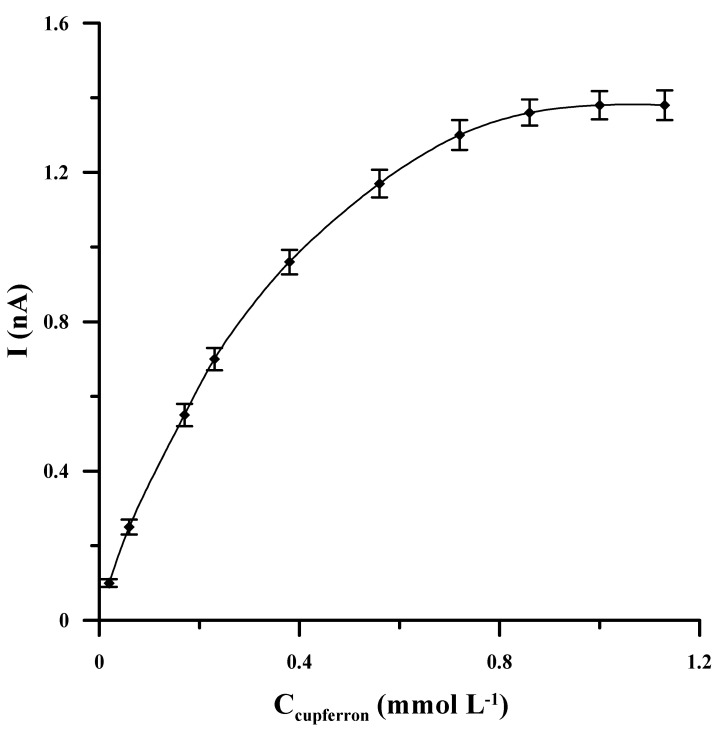
The effect of cupferron concentration on the AdSV voltammetric response of 1 × 10^−8^ mol L^−1^ V(V). The accumulation potential and time were −0.4 V and 60 s, respectively.

The basis of the proposed procedure is the adsorptive preconcentration of vanadium in the form of a complex with cupferron. Therefore, without the addition of cupferron, no stripping peak was obtained by recording the voltammogram from 0.1-mol L^−1^ acetate buffer (pH = 4.6) containing 1 × 10^−8^-mol L^−^^1^ V(V) without the addition of cupferron. It was observed that the lowest concentration of the complexing agent at which the vanadium peak appeared was 2 × 10^−5^ mol L^−1^, while a further increase in the cupferron concentration to 1 × 10^−3^ mol L^−1^ led to an enhancement in the voltammetric response of vanadium. However, when the concentration of cupferron in the analyzed solution was higher than 1 × 10^−3^ mol L^−1^, no further changes in the height of the vanadium peak were observed (Figure 2), so the concentration of 1 × 10^−3^ mol L^−1^ was chosen as the most favorable in the vanadium determination.

### 3.3. Optimization of Activation Potential and Time

During the optimization studies, it turned out that a much better-shaped vanadium peak was obtained by preceding the preconcentration step by applying a high negative potential value to the electrode. At that time, the electrode surface was activated, so it was cleaned of the residues from the previous measurements. When analyzing the influence of the activation potential (whose value was changed in a range from −2.5 to −2.1 V) on the vanadium signal, it was found that the lower the activation potential value was applied to the electrode surface, the better the voltammetric response was obtained. It was also proven that the shorter the activation step, the higher the V(V) peak current appeared in the voltammogram. Comparing the height of the vanadium signal for the activation time varying within a time interval from 1 to 5 s, it was shown that the most advantageous activation of the electrode surface was obtained using a potential of −2.5 V for 2 s.

### 3.4. Optimization of Accumulation Potential and Time

**Figure 3:**
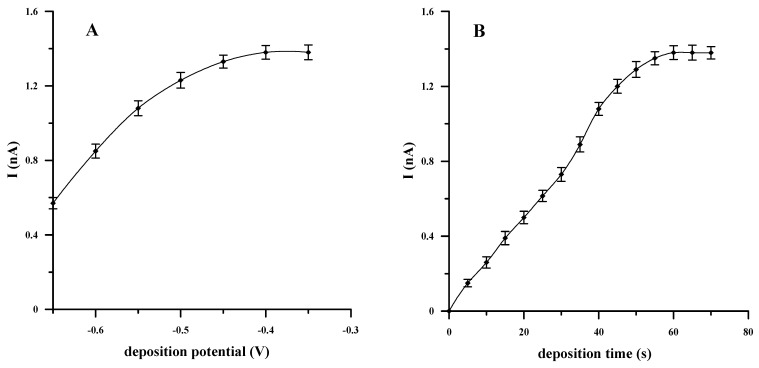
The effect of the accumulation potential (**A**) and its time (**B**) on the AdSV voltammetric response of V(V). Fixed concentration: 1 × 10^−8^ mol L^−1^ V(V), 0.1 mol L^−1^ acetate buffer (pH = 4.6) and 1 × 10^−3^ mol L^−1^ cupferron.

The effect of the accumulation potential on the voltammetric response of V(V) was tested over the potential range of −0.65 to −0.35 V (every 0.05 V) for a fixed accumulation time equal to 60 s using a solution that contained 1 × 10^−8^-mol L^−1^ V(V), 1 × 10^−3^-mol L^−1^ cupferron and 0.1-mol L^−1^ acetate buffer (pH = 4.6). As shown in Figure 3A, the V(V) peak current increased, with the potential varying from −0.65 to −0.40 V, while, subsequently, in a range from −0.40 to −0.35 V, the stripping peak current remained unchanged. Therefore, a potential equal to −0.4 V was used in all experiments.

The optimization studies also included a selection of the accumulation time at which the adsorption of V(V)–cupferron complexes on the electrode surface was the most efficient. The effect of the accumulation time was investigated by recording the V(V) signals for various accumulation times, extending the time from 5 to 70 s every 5 s. It was noted that extending the time of the accumulation step to 60 s resulted in an increase in the V(V) peak current. On the other hand, extending the accumulation time from 60 to 70 s did not improve the voltammetric response, which remained unchanged. (Figure 3B). The amplification of the voltammetric signal with increasing the accumulation time is related to the fact that more complexes are able to adsorb onto the surface of the working electrode. Nevertheless, at some point, despite an increase in the accumulation time, the signal height becomes constant as a result of complete saturation or equilibrium of the surface coverage [22]. Given the above, the optimal circumstances adopted in the accumulation step for the determination of V(V) are as follows: a potential of −0.4 V and a time of 60 s.

### 3.5. Characteristics of the Analytical Procedure

After applying all the above-optimized measurement parameters, the linearity of the calibration curve was maintained in a range from 8 × 10^−10^ to 1 × 10^−7^-mol L^−1^ V(V) with the regression equation of y = 148.49x − 0.099, where y: the peak’s current/nA and x: concentration/µmol L^−1^ (R^2^ = 0.999).

The detection limit (LOD) evaluated based on three times the standard deviation of the blank response divided by the slope of the calibration curve was equal to 2.5 × 10^−10^ mol L^−1^. For comparison, the LOD calculated according to the upper limit approach was 7 × 10^−10^ mol L^−1^ [23]. The quantification limit (LOQ) evaluated based on ten times the standard deviation of the blank response divided by the slope of the calibration curve was equal to 8.3 × 10^−10^ mol L^−1^. The repeatability of the recommended procedure was evaluated based on eight consecutive measurements for solutions comprising 2 × 10^−9^-mol L^−1^ V(V), whereas the relative standard deviation was found to be 3.4%. The reproducibility of the method was established in accordance with the measurement results for the solutions comprising 2 × 10^−9^-mol L^−1^ V(V) obtained over 5 consecutive days, whereas a relative standard deviation of 5% was obtained. The long-term stability of the solid bismuth microelectrode was examined after two and six months of its usage, and it turned out that the vanadium signal retained 95% and 92%, respectively, of the initial voltammetric response.

Table 1 compares the analytical parameters of the proposed procedure with other procedures developed to date with the use of adsorptive stripping voltammetry (AdSV). As shown, most of these procedures are based on the use of mercury electrodes [7,8,9,10,11,12,13,14,15,16]. For the determination of vanadium, a mercury-free black acetylene paste electrode (ABPE) [17], bismuth film electrode (BiFE) [18] and lead film electrode [19] have also been used. All procedures are arranged according to the decreasing detection limit. As can be seen, the lowest detection limit (9 × 10^−12^ mol L^−1^) is achieved in the procedure developed with the use of the HMDE electrode [9]. On the other hand, if we compare the proposed procedure to other methods based on mercury-free electrodes, it can be seen that the procedure recommended by us is characterized by the lowest limit of detection (2.5 × 10^−10^ mol L^−1^) among them, and it has the same broad linearity range as in Reference [17], covering more than two orders of magnitude (8 × 10^−10^–1 × 10^−7^ mol L^−1^). Taking into account the duration of the preconcentration step, it can be seen, in most procedures, the time of the accumulation stage was greater than or equal to 90 s, while, in the proposed procedure, the accumulation stage was shorter and amounted to 60 s. 

### 3.6. Tolerance to Interfering Species

The impact of coexisting cations and organic compounds on the quantification of V(V) was precisely examined. Many aquatic cations (Au(III), Bi(III), Cd(II), Co(II), Cr(III), Cu(II), Fe(III), Ga(III), Hg(II), In(III), Mn(II), Mo(VI), Ni(II), Pb(II), Pt(IV), Sb(III), Sn(II), Ti(IV), Tl(I), W(VI) and Zn(II)) were investigated as the possible interferents. The concentrations of these ions were changed in the range of 1 × 10^−8^ to 1 × 10^−5^ mol L^−^^1^, whereas the concentration of V(V) in the tested solution was 1 × 10^−8^ mol L^−1^. It turned out that the height of the voltammetric peak of vanadium was in the range from 95% to 105% after adding the most of the examined interferents (in the whole tested concentration range) in comparison to the voltammetric response obtained in the presence of vanadium alone. This is a great advantage of the developed procedure [19], especially the maximum permissible concentrations of foreign ions such as Cd(II), Cr(III), Fe(III), Ni(II) and Mo(VI), which do not affect the V(V)–cupferron response at a lead film electrode, which are lower or equal to 2 × 10^−6^ mol L^−1^ for Cd(II), Cr(III), Fe(III) and Ni(II) and 5 × 10^−7^ mol L^−1^ for Mo(VI). Furthermore, in the proposed procedure, upon addition of the cations such as Pb(II), Ti(IV) and W(VI) to the tested solution, an additional peak appeared in the vicinity of the V(V) signal in the voltammogram while leading to a slight reduction in the V(V)–cupferron response by 10, 22% and 15%, respectively, at the highest tested interferent concentration equal to 1 × 10^−5^ mol L^−1^, compared to the V(V) peak current without the addition of the interfering substances.

The proposed procedure was also tested for sensitivity to the presence of organic substances such as Triton X-100 and humic acids (HA) as representatives of surface active and humic substances, respectively. The research was carried out in a solution containing vanadium(V) at a constant concentration equal to 5 × 10^−9^ mol L^−1^ and organic substances, the concentrations of which varied in **a** range from 0.1 to 2.0 mg L^−1^. The research shows that, in the concentration range from 0.1 to 0.4 mg L^−1^, none of the tested organic substances interfered with the determination of vanadium. However, a concentration of 0.5-mg L^−1^ Triton X-100 and HA reduced the voltammetric response of V(V) by about 20 and 45%, respectively. At the highest tested concentration of both organic substances, the height of the vanadium peak was 25% of the height of the signal recorded without the presence of organic substances. This is a very satisfactory result, considering that, in others works concerning the determination of V(V), in which the effects of both Triton X-100 and HA were investigated, it was noted that in a concentration range from 0.2 to 1 mg L^−1^, they caused the total vanadium signal to fade [15,16,19].

### 3.7. Verification of the Procedure Using SPS-WW1 Waste Water CRM and Real Water Sample

**Figure 4:**
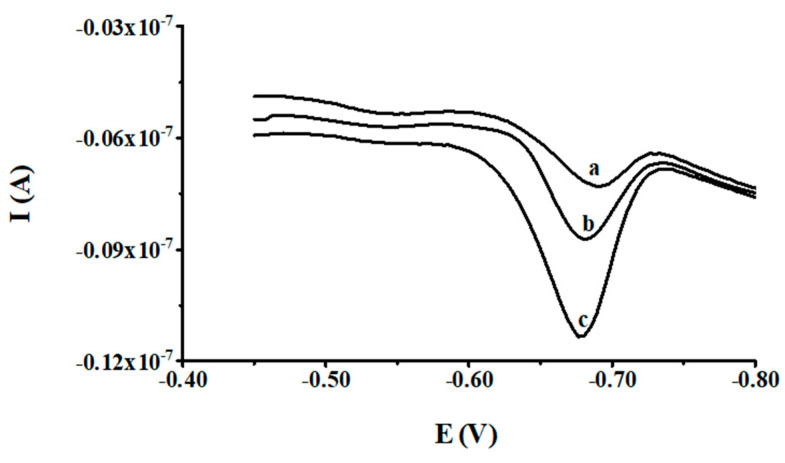
The AdSV voltammograms obtained during vanadium quantification in the certified reference material SPS-WW1 waste water: SPS-WW1 diluted (1:200) (a) as (a) + 1 × 10^−8^ mol L^−1^ V(V) (b) and as (a) + 2.5 × 10^−8^ mol L^−1^ V(V) (c).

In order to assess the applicability, the developed AdSV procedure was applied to analyze the certified reference material SPS-WW1 waste water (series 111). Besides its complicated organic matrix, this material has a rich elemental composition confirmed by a certificate. The total elemental composition of this material is presented in Table 2. The vanadium content of CRM was determined by diluting it with the supporting electrolyte using the standard addition method in the proposed procedure under optimum conditions. As a result of the vanadium determination in the tested CRM using the developed procedure, a concentration equal to 95.1 ± 1.6 ng mL^−1^ was obtained. This was an average value derived from three measurements, which was in a good agreement with the certified concentration (100.0 ± 0.5 ng mL^−1^). Thus, it was shown that our procedure is suitable for the analysis of environmental water samples with a complex matrix. Exemplary voltammograms recorded in the course of quantification of V(V) in the certified reference material SPS-WW1 waste water are depicted in Figure 4.

Furthermore, natural water samples such as water from the Ciemiega River, rain water and tap water, all spiked with vanadium(V), were analyzed by the proposed procedure. The results from the V(V) quantification are presented in Table 3. The recovery values between 96% and 106% for V(V) with RSD ranging from 3.1% to 4.2% are evidence of an acceptable accuracy of the proposed method and clearly testify the applicability of this procedure for V(V) determination in various real water samples.

## 4. Conclusions

This work is the first one to report the use of an environmentally friendly solid bismuth microelectrode for determination of a trace V(V). This method is characterized by the following features:

1. The method is simple to implement and makes the analysis eco-friendly, as it allows avoiding the following:
  - the use of metallic mercury for electrode formation (thus, the measurements can be carried out without aerating the electrolyte with an inert gas);
  - adding bismuth ions to the voltammetric cell when using a film bismuth electrode (therefore, the procedure is considerably simplified and allows for a lesser consumption of reagents).
2. The procedure shows a very good analytical performance, such as:
  - a low detection limit of 2.5 × 10^−10^ mol L^−1^ (60 s);
  - a broad linearity range covering more than two orders of magnitude (8 × 10^−10^ to 1 × 10^−7^ mol L^−1^);
  - good repeatability and reproducibility with the calculated RSD = 3.4% (*n* = 8) and 5.0% (*n* = 5), respectively.
3. In the recommended procedure, the height of the V(V) signal is unchanged in the presence of an even 1000-fold excess of many foreign ions in the sample.
4. This method makes it possible to carry out vanadium determinations in the presence of 0.5-mg L^−1^ Triton X-100 and humic acid, while, in other procedures, no signal of V(V) was obtained at the concentration of organic substances.
5. During the verification of the procedure using a certified reference material and real water samples, it was shown that the developed procedure is suitable for the analysis of environmental water samples with a complex matrix, even waste water.

## Figures and Tables

**Table 1:** The comparison of the analytical parameters of the proposed procedure with the other AdSV procedures developed to date.

Working Electrode	Complexing Agent	Detection Limit (mol L^−1^)	Linear Range (mol L^−1^)	Accumulation Time (s)	Analytical Application	References
HMDE	chromoxane cyanine R	$1.0\;\times \;10{}^{-7}$	3.0 × 10^−7^–2.4 × 10^−5^	180	food samples, tap water, rainwater	[12]
MFE	pyrogallol	$2.0\;\times \;10{}^{-8}$	$\mathrm{ND}–1.5\;\times \;10{}^{-6}$	180	certified reference materials	[15]
BiFE	chloranilic acid	$3.9\;\times \;10{}^{-9}$	$9.8\;\times \;10{}^{-8}$ $–5.0\;\times \;10{}^{-7}$	600	groundwater	[18]
ABPE	alizarin violet	$6.0\;\times \;10{}^{-10}$	$8.0\;\times \;10{}^{-10}$ $–1.0\;\times \;10{}^{-7}$	90	environmental water	[17]
PbFE	cupferron	$3.2\;\times \;10{}^{-10}$	$1.0\;\times \;10{}^{-9}$ $–7.0\;\times \;10{}^{-8}$	30	certified reference materials, river water	[19]
BiFµE	cupferron	$2.5\;\times \;10{}^{-10}$	$8.0\;\times \;10{}^{-10}$ $–1.0\;\times \;10{}^{-7}$	60	certified reference material, river water, rain water, tap water	this work
HMDE	cupferron	$2.0\;\times \;10{}^{-10}$	$2.0\;\times \;10{}^{-9}$ $–2.0\;\times \;10{}^{-6}$	50	food samples, mineral water, environmental water	[7]
HMDE	dihydroxybenzaldehide	$2.0\;\times \;10{}^{-10}$	$5.0\;\times \;10{}^{-10}$ $–5.0\;\times \;10{}^{-8}$	30	environmental water	[13]
MFE	cupferron	$1.6\;\times \;10{}^{-10}$	$2.0\;\times \;10{}^{-9}$ $–6.9\;\times \;10{}^{-8}$	90	sea water	[14]
HMDE	cupferron	9.0 × 10^−11^	$1.0\;\times \;10{}^{-8}$ $–9.0\;\times \;10{}^{-7}$	ND	ND	[8]
HMDE	catechol	7.0 × 10^−11^	ND	120	sea water	[10]
HMDE	dihydroxynaphthalene	1.5 × 10^−11^	$5.0\;\times \;10{}^{-11}–4.0\;\times \;10{}^{-9}$	60	environmental water	[11]
Hg(Ag)FE	chloranilic acid	1.0 × 10^−11^	$2.5\;\times \;10{}^{-10}$ $–1.0\;\times \;10{}^{-7}$	90	certified reference materials, tap water	[16]
HMDE	chloranilic acid	9.0 × 10^−12^	$2.0\;\times \;10{}^{-10}$ $–5.0\;\times \;10{}^{-8}$	100	certified reference materials	[9]

ND—no data, HMDE—hanging mercury drop electrode, MFE—mercury film electrode, BiFE—bismuth film electrode, ABPE—acetylene black paste electrode, PbFE—lead film electrode, BiFµE—solid bismuth microelectrode and Hg(Ag)FE—renewable mercury film silver-based electrode.

**Table 2:** Concentrations of the elements in the certified reference material SPS-WW1 waste water.

Element	Concentration in ng mL^−1^ (20 °C)
Al	2000 ± 10
As	100.0 ± 0.5
Cd	20.0 ± 0.1
Co	60.0 ± 0.3
Cr	200 ± 1
Cu	400 ± 2
Fe	1000 ± 5
Mn	400 ± 2
Ni	1000 ± 5
P	1000 ± 5
Pb	100.0 ± 0.5
V	100.0 ± 0.5
Zn	600 ± 6

**Table 3:** Salvage values of V(V) obtained from spiked real water samples using the AdSV method.

Sample	Concentration of Added V(V) (nmol L^−1^)	Concentration of Found V(V) (nmol L^−1^)	Recovery (%)	RSD (*n* = 5) (%)
Ciemiega river water	5 10	5.2 9.9	104 99	3.1 3.7
Tap water	5 10	4.9 9.6	98 96	3.8 4.0
Rain water	5 10	5.3 10.4	106 104	4.2 3.3

## Data Availability

Not applicable.
